# A Study of DNN-Based Media Literacy and Distance Education Management System in the Context of Participatory Culture

**DOI:** 10.1155/2022/3666274

**Published:** 2022-05-20

**Authors:** Rui Xu, Chen Wang, Yen Hsu

**Affiliations:** ^1^The Graduate Institute of Design Science, Tatung University, Taipei 104, Taiwan; ^2^School of Art and Design, Fuzhou University of International Studies and Trade, Fuzhou 350200, China; ^3^College of Journalism and Communications, Shih Hsin University, Taipei 11604, Taiwan

## Abstract

Through an in-depth study and analysis of the integration of media literacy with distance education management using the DNN algorithm in the context of participatory culture, and the design of a distance education management system for application in actual teaching, the word embedding model is used to embed the ratings and tags, respectively; then, the self-encoder is used to extract textual features for item tags, while DNN is used to extract features for user tagging behaviors; finally, the fully connected neural network is used to predict ratings for the fused user and item features. The experimental results show that the optimized recommendation model effectively reduces the user rating prediction error while alleviating the cold start problem. A blockchain-based learning platform is designed. The purpose is to solve the contradiction between centralized storage and social trust, and to transfer the management of educational certification resources from centralized control management to distributed control management. This platform can record in stages a variety of verification information and course certificate information collected by students in the process of learning courses, create course certificates containing digital summaries of learning information, and store them on the chain to ensure the integrity and traceability of data in the learning process. This paper implements a blockchain-based online education system, which ensures that system data will not be maliciously queried and dynamically changed by management.

## 1. Introduction

Web technology offers us many possibilities, not only as a tool but also as a glue for multiple relationships in society. After Web 2.0 technologies were developed, several new media such as wikis, various social media, video, and audio sites, a new era of more interactive and participatory media began to arrive. The role of web technologies in the system of information and knowledge production and dissemination has received much attention. The space for people to produce, consume, and disseminate is gradually shifting to such a network space [1]. While most traditional media are in a linear model of communication, with feedback and interaction but not to a high degree, the form of communication on the Internet is more flexible, the autonomy of all people is enhanced, the degree of participation is increased, and the identity of the transmitter and the recipient is no longer unique, but one can be both. Participants are no longer limited to being consumers or producers of information resources but can be both. It is also because the mechanism of participation has become more flexible that the production of knowledge in the media has become more contemporary. Social development varies from one social environment to another, and people's practice concepts and ways of thinking also have unique characteristics of each era [2]. With the rapid development of media technology, traditional media continue to seek new paths for themselves in the Internet environment, and the process of change in broadcast media also presents several different stages of development; in each stage, technology and ideas are constantly colliding, making it present a unique development trend. It is also because of the changing environment that, in the era of mobile Internet, broadcasting has become more flexible in form and stronger in the sense of subject participation, especially in mobile audio, which is more reflective of several features of participatory culture.

However, during the long development process of radio, media managers are constantly figuring out how to better serve their listeners [3]. In terms of the use and dissemination of radio technology, communicators are using increasingly rapid ways to deliver information, thanks to the constant development of media technology with a material base of assurance. The concept of paying attention to audience needs can also be reflected in the changes in the form of radio content. In the period of deepening the reform of radio and television, the targeting of central and local radio stations is a requirement put forward by the central government. And in the face of different audiences, the content is launched to meet their needs, while the diversification of column settings, news, economy, culture and social education, ethnic minorities, and Hong Kong and Taiwan propaganda multiple settings are reflected in their professionalism and relevance, and the needs of audiences of various attributes can be treated correctly [4]. In different social environments, the forms of social development are different, and people's practical concepts and ways of thinking also have the unique characteristics of each era. With the development of economic globalization, to meet the new challenges and the new situation facing the current social development, many countries attach great importance to education, through education, improve the quality of the nationals, improve scientific research capabilities, and increase national comprehensive strength. Fundamentally, this is the competition of talents. To cultivate more excellent talents, the level of development of network teaching technology has been continuously improved and good results have been obtained. For the time being, developed countries such as Europe and America are at the forefront of the world in terms of the high level of development in online distance education and have accumulated a lot of experience. Online distance education has made great progress and rapid development, among which online distance education for primary and secondary school students occupies a relatively large proportion.

Under the background of rapid development of Internet technology, the traditional education model has exposed many drawbacks, which directly affect the improvement of the actual teaching level. Therefore, to meet the new changes and demands arising from social and economic development and teaching mode, schools should base on their actual situation, introduce advanced teaching concepts, and set up professional integrated network distance learning management systems to meet the basic requirements of current distance education. However, according to the current situation of distance education, there are still many problems that cannot meet the basic requirements of the current rapid development of education. In the future development process of network distance learning management systems, researchers need to integrate more advanced education concepts, continuously enrich the system functions, and actively develop functional modules for sex, to promote the benign development of the current distance learning industry. New needs give rise to new solutions, and this solution must allow us to have an autonomous and transparent future. In this solution, information technology must exist to ensure that learning achievement data cannot be falsified or tampered with and that this data can be authenticated and stored in a distributed manner by multiple subjects, and finally, the authority of strong and weak authorities can be balanced within the same alliance, so that the alliance can have the credibility of an authoritative subject while being flexible enough to apply to market demand. Blockchain technology, which has been practiced for ten years, has just such a capability. It can not only solve the problem of the insufficient authority of online education enterprises by introducing strong authority nodes, but also enhance the credibility of the enterprises through the nontamperable learning certificates.

## 2. Related Works

In a public space like mobile audio, the traditional quasi-social relationship turns into a social relationship, and the participants become countless nodes in the media relationship, and the mode of communication and interaction becomes many-to-many, no longer limited to listeners and a certain presenter. Participants are in constant contact with the subjects, contents, and technologies in the space. They no longer wait for some presenter to guide them to participate in the audio content, but personally take the initiative to listen, produce, and disseminate the content based on their own experience of using it [5]. Fundamentally, this is a competition for talent. To cultivate more outstanding talents, the development level of online teaching technology has been continuously improved, and good results have been obtained. The establishment of a new type of distance education system plays a very important role in improving the quality of education and promoting the future of the education industry. To do a good job in distance education, the Open University of the UK has set up corresponding regional offices in each city, established hundreds of learning centers, adopted a vertical approach to the management of the running distance higher education system, formed a government-led management approach, coordinated the relationship before the top and bottom, ensured timely feedback of information, rationalized the distance education process, and improved the efficiency of actual education [6].

The performance of the network distance learning system has been continuously improved, forming a large-scale development, but many projects carried out still have shortcomings and need to be further improved. Firstly, from the current network distance learning system, there are many problems in teaching content and teaching form, which are still influenced by the traditional teaching model, or even simply copying and reproducing the original knowledge content, which cannot improve the actual teaching quality, the learning enthusiasm is not high, and the advantages of distance learning cannot be given full play [7]. Secondly, the development of distance learning content is not strong enough [8]. From the current online distance learning content, the quality of distance learning resources needs to be improved, and the type of teaching courses is single, which cannot effectively meet the needs of learners in many aspects [9]. After participating in distance learning, learners cannot get a timely and effective evaluation, and there are fewer opportunities for online interaction so they cannot communicate timely and effectively for the problems in learning, which affects the teaching effect [10]. Finally, the degree of sharing of distance learning resources is not high. In the process of designing courseware for the current online distance learning platform, the lack of corresponding norms and standards cannot meet the individual needs of learners, especially the lack of independent learning courseware, which makes it difficult to realize the sharing of high-quality teaching resources and has an extremely negative impact on the current distance education [11].

The teacher user who publishes the course will verify the digital signature attached to the application when reviewing the certificate application and will review the student's application information only after it is approved [12]. Once the application is approved, the teacher will use his or her private key to digitally sign the student's complete learning record information. However, according to the current situation of distance education, there are still many problems, which cannot meet the basic requirements of the current rapid development of education. The alliance chain will introduce strong authority witness nodes, combined with weak authority business nodes built by online education enterprises, and use the authority proof mechanism to balance the authority of strong authority subjects and weak authority subjects within the same alliance chain, so that the alliance chain can share the credibility of strong authority subjects, but also be flexible enough to apply to market demand. Using the natural trust mechanism of blockchain technology to solve the trust problem of the existing online education industry is the application direction of this paper.

## 3. Design of Media Literacy and Distance Education Management System for Participatory Culture DNN

### 3.1. Analysis of DNN Fusion Media Literacy Algorithm

The method uses two neural network modules to simulate the processing of the DNN algorithm, representing the processing of the real part of its SCF algorithm in the time domain and the imaginary part in the time domain, respectively. Each neural network module consists of one input layer, two hidden layers, and one output layer. The first hidden layer has one neuron and the second hidden layer has two neurons. The learning algorithm uses the Levenberg Marquardt algorithm, which is like the DNN algorithm after training and reduces the computational complexity significantly [13]. For an OFDM system with N subcarriers, there is no need to add additional DNN calculations; only the addition of 10N real numbers and the multiplication of 10N real numbers are required.  Table 1 gives a comparison of the complexity of the neural network-based implementation of SCF with the original SCF algorithm for the parameter settings: the number of OFDM subcarriers is 256 and the number of iterations is 3.

**Figure 1:**
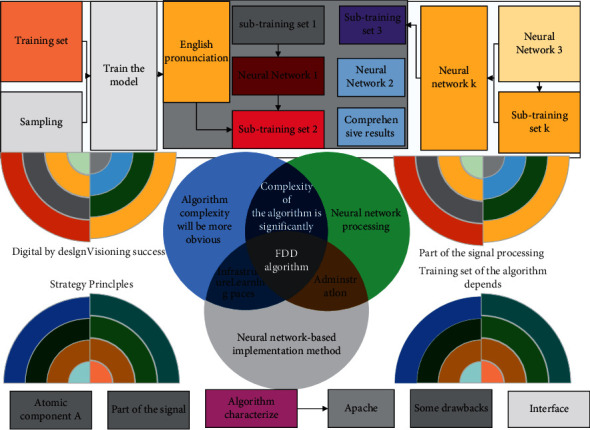
Framework of DNN fusion media literacy algorithm.

The complexity of the algorithm is significantly reduced by neural network processing. And as N increases, the difference in algorithm complexity will be more obvious, and the neural network-based implementation method has obvious advantages. However, the algorithm also has some drawbacks. The two neural network modules of the algorithm characterize the time-domain real part of the signal and the time-domain imaginary part of the signal processing, and the training set of the algorithm depends on the SCF algorithm, which does not separate the real part of the signal from the imaginary part in the process of processing OFDM signals; i.e., there is a certain connection between the real part of the signal and the imaginary part of the signal processing. Then, use many signals processed by the SCF algorithm prepared in advance for training. The learning algorithm adopts the Levenberg–Marquardt algorithm. After training, the effect like the DNN algorithm can be achieved. And the algorithm completely separates the two modules from each other in the process of training the neural network, making the real and imaginary parts of the signal completely separated from each other, and for some specific cases, there is a problem of mapping confusion, as in Figure 1.

There are two hidden layers, each of which contains a fully connected layer, a bias term, and a Tanh activation function, and the number of neurons in the fully connected layer can be self-determined. The output layer contains one neuron, one bias term, and a linear activation function. The output layer contains a neuron, a bias term, and a linear activation function. Then, the input-output correspondence of this neural network is

(1) $$\begin{array}{c} NN_{r}\left(x_{\text{train}}\right)=W_{3}\left(\text{tamh}\left(W_{2}\left(\tanh\left(W_{1}\left(x_{\text{train}}\right)-b_{1}\right)-b_{2}\right)-b_{3}\right)\right). \end{array}$$

In addition to the disadvantage of mapping confusion, the traditional neural network structured OFDM system suffers from spectrum leakage. Since the neural network-based CM scheme for suppressing OFDM signals is still processing the signal in the time domain, there exists an out-of-band leakage of the signal due to the distortion of the signal in the time domain, which interferes with the frequency band. To improve the comprehensive performance of the system, filtering will be performed after the neural network processing.

(2) $$\begin{array}{c} h_{1}=\sigma_{e}\left(W_{1}x-b_{1}\right),\\ y=\sigma_{e}\left(W_{2}h-b_{2}\right). \end{array}$$

Further analysis shows that, for the neural network, the essence of its operation is matrix operation, and the neural network does not distinguish between the real and imaginary parts of the signal, so the processing of the neural network for the real and imaginary parts of the signal processing is identical, and the parameters of its fully connected layer are possible to share. To further simplify this network structure, the two neural network modules of this improved algorithm are reduced to one neural network module, and the input and hidden layers of this module remain unchanged, and one neuron of the output layer is changed to two neurons, corresponding to the real and imaginary parts of the output signal, respectively.

The encoding phase can be viewed as a deterministic mapping converting the input signal into the hidden layer expression, while the decoding phase is to try to remap the hidden layer expression into the input signal. By minimizing the objective function, one can learn to obtain the weights and biases in a conventional self-encoder. In addition to the mean square error given in (3), the loss function can also be chosen as the cross-entropy, which is expressed as follows:

(3) $$\begin{array}{c} J\left(W,b\right)=\sum_{i=1}^{n}\left(x_{i}\ln\left(y_{i}\right)-\left(1-x_{i}\right)\ln\left(1+y_{i}\right)\right). \end{array}$$

During the frequent use of mobile audio, some shifts in people's inherent views develop. Learning relevant knowledge through audio programs, which is a breakthrough from the original offline education, is also different from online video education; not only is the variety of learning richer, but also the cost and form of learning are different, making people master the knowledge they need within a limited time and money into control. After all, hearing is a weak storage system, and it is a challenge to deep rational thinking because it is fleeting and cannot be explored deeply.

The hidden layer of the traditional self-encoder has 3 different forms, namely, compressed structure, sparse structure, and equal-dimensional structure [14]. When the number of neurons in the input layer is larger than the hidden layer, it is called a compressive structure; when the number of neurons in the input layer is smaller than the hidden layer, it is called a sparse structure; if the number of neurons in the input layer and the hidden layer is equal, it is an equal-dimensional structure.

In a conventional self-encoder, the weights of encoding and decoding stages are trained separately and there is no connection between them, but if there are the same weights between encoding and decoding, such encoder is called a bound weight self-encoder (TAE). For the conventional self-encoder, the degree of weight reduction can also be controlled by adding a weight decay term to the loss function, and this self-encoder is called the conventional regular self-encoder with the loss function as follows:

(4) $$\begin{array}{c} J_{CoAE}\left(W,b\right)={\sum \left(L\left(x,y\right)\right)-\lambda \left\|W\right\|}^{3}. \end{array}$$

Of course, the loss function alone is not enough; the neural network also needs to be parameterized to minimize the loss function, which is the actual meaning of “training.” Minimizing the loss function means that the closer the predicted value of the neural network is to the expected value in the training set, the better the fit of the neural network will be. When the number of neurons in the input layer is smaller than that in the hidden layer, it is called a sparse structure; if the number of neurons in the input layer and the hidden layer is equal, it is an equal-dimensional structure. Therefore, how to adjust the parameters of the neural network is also one of the key issues. In general, using the gradient descent class is the most effective and straightforward method. In this paper, the Levenberg–Marquardt algorithm of the gradient descent method will also be used to train the neural network.

(5) $$\begin{array}{c} \mu =\frac{1}{T}\sum_{t=0}^{T-1}y_{t}^{2}. \end{array}$$

The mean vector is subtracted from all the feature frame vectors to obtain the CMN processed feature vectors.

(6) $$\begin{array}{c} \mu =\frac{1}{M}\sum_{m=0}^{M-1}y_{m}^{2}. \end{array}$$

**Figure 2:**
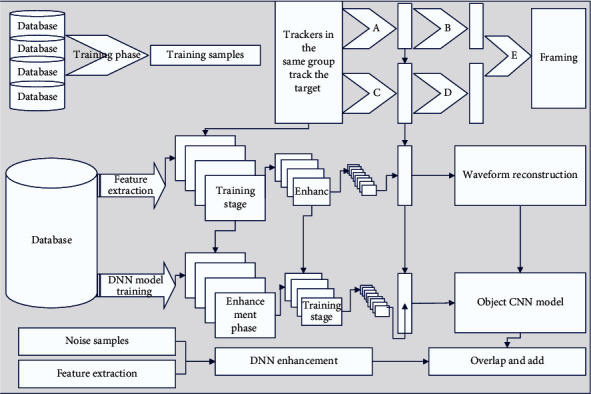
Block diagram of augmentation model based on deep neural network.

When inertia coefficients are applied, the model update is related to not only the current gradient, but all previous gradients, thus increasing the convergence speed. In practice, if the error surface has very small points, the obtained gradient will be disturbed and fluctuate continuously, and the inertia coefficient can be used to alleviate this problem and obtain a faster training speed, as shown in Figure 2.

The reason for using the DNN model in this paper is that the deep structure and nonlinear simulation ability of DNN can learn the complex relationship between clean speech and noisy speech very well [15]. To make the DNN model generalize better, many lines of the corpus are constructed in this paper, which is mainly obtained by adding two by two according to the additive model.

This is because the DNN model itself can obtain contextual information between neighboring frames and the information of each dimension in the whole frequency band, and it can apply this information in the time and frequency domains very well. This is demonstrated by its ability to concatenate all information into a long input feature vector to be fed into the DNN model for learning, allowing the model to learn the complex relationships between clean and noisy speech.

The fitness function is used to calculate the fitness of the current individual, evaluate the chromosomal merit, and directly affect the probability that the individual will be selected in the population. Therefore, the fitness function determines the evolutionary direction of the population and the reasonableness of the optimal solution derived by the algorithm. Moreover, for the sake of computational simplicity, the fitness function should be designed to be as simple as possible, and the calculated fitness value must be positive to ensure that individuals are selected according to their degree of superiority or inferiority and that less memory and computational resources can be used for the calculation of the fitness.

The degree of willingness in the morning was higher than in the afternoon and higher than in the evening, which is related to the fact that students have more active logical thinking in the morning. Although the first two periods in the morning are slightly lower compared to the later ones, this is related to the subjective factor that a small number of students do not want to get up early [16]. In traditional autoencoders, the weights of the encoding and decoding stages are trained separately, and there is no connection between the two, but if there is the same weight between encoding and decoding, such an encoder is called a bound weight autoencoder device. The table also shows that the priority of students for theory classes is decreasing from Monday to Friday, which means that the first half of the week is more efficient than the second half of the week. For Friday, both theoretical and experimental courses need to be considered for vacation.

### 3.2. Participatory Cultural Distance Education Management System Design

In this paper, we adopt the idea of separating the front and back ends to design. The back-end part is responsible for the model and controller part of the MVC pattern, mainly focusing on business logic and data processing; the front-end part is responsible for the interface display and front-end event processing. The representation layer is mainly responsible for interface display and interaction experience, so the popular React front-end framework is adopted directly [17]. Meanwhile, the front-end part of the create-react-app scaffold-based development is responsible for handling the logic of the representation layer.

The system described in this paper requires a total of 11 business functions according to business requirements, including two each for storage, query, and application. The key data of the system such as certificate data and school registration data are stored in the upper chain to ensure the authenticity of the data. Most of the system functions are completed through front- and back-end interaction. Except for the registration operation, all operations must be performed under the login status. At the same time, the study time, vignette assignments, and examination results in the study course are required as reference data when applying for the course certificate. Student users need to note that there are two prerequisites for applying for credit: one is whether they have obtained the certificate of completion of the course for which they apply for credit, and the other is whether there exists verifiable academic information [18]. Only after passing these two audits will the system notify the student's credit application to the corresponding university user.

**Figure 3:**
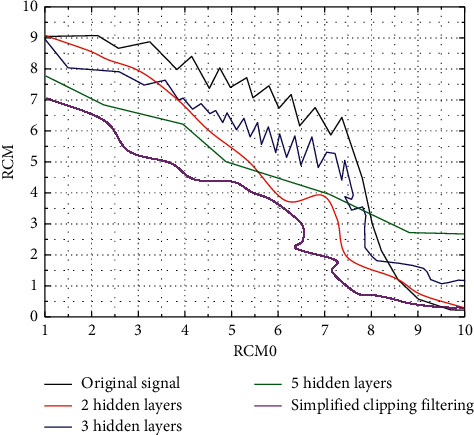
CCDF performance with the same number of hidden layers.

Users need clear permission verification in the process of using this design system, which can be based on the basic requirements of the operation, and students can select the corresponding courses according to the actual situation and then manage the course grades. And in the whole grade interface, you can check your study results, and if you have doubts about your grades, you can apply to check your grades. But ordinary users cannot make changes to their grades [19]. The system selects the corresponding course selection grades from different areas and then transmits the course selection grade management information to the database in the background, as shown in Figure 3.

After writing the contract, the compile JS script is used to compile the smart contract, and the compile result is written to a JSON file with the same name as the contract file, and then the bytecode key corresponding to the hex value is deployed to the federated chain platform. The real-time recommendation provides corresponding data support, which relieves the pressure of real-time computing on the server. Once the system is started, users can use the Metalmark plugin for Chrome to invoke some of the smart contract's functions from their browser. The corresponding administrator information management Error Guessing Testing relies entirely on experience, and the test cases it designs are what we often call guesses, where the system senses an error and then tests the relevant cases. If we want to get the corresponding test cases, we will quickly design the test cases; the software in a certain aspect of the realization will be wrong, and the advantage is fast; if you want to get, it is all by the experience and knowledge accumulated in the actual work.

By using many noisy speeches with labels to train the model to determine whether the speech after framing is a speech frame or not, our proposed model is improved mainly from two aspects: training data and model structure. In terms of training data, we train the model using the speech endpoint detection (VAD) technique, do processing on the training data, divide the training set into speech segments and nonspeech segments, and then train the two training sets separately [19]. To improve the generalization ability of the model, we added 100 different types of noise in the training set to improve the model's ability to handle nonsmooth noise.

**Figure 4:**
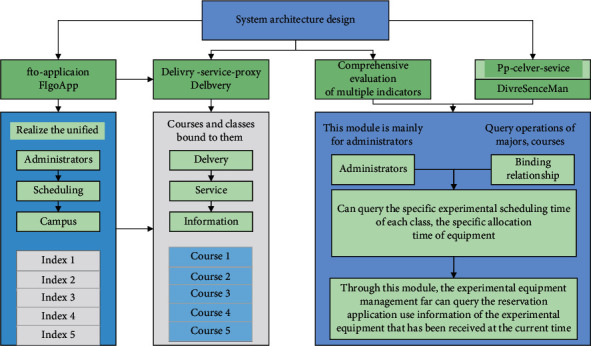
System architecture design.

Although the simplified limit filter has low complexity, its algorithm causes the most serious signal distortion, which is reflected by the worst performance of BER [20]. The iterative limiting filter, the conventional neural network, and the integrated learning-based neural network proposed in this paper all have good BER performance, and their performance is generally consistent. In the case of BER of 10–5, the Eb/N0 of the simplified limiting filtering algorithm is about 10.8 dB, while the Eb/N0 of other algorithms is about 9.8 dB, which is about 1 dB higher than the simplified limiting filtering algorithm, as shown in Figure 4.

This module is mainly for administrators to add, change, delete, and query operations of majors, courses, and classes, as well as the binding relationship between administrators [21]. Teacher users can query the courses and classes bound to them according to this page to realize the unified course arrangement. This module is mainly for administrators or teachers who are responsible for daily scheduling management on campus. Through the imported daily course information, the system realizes automatic allocation of experimental course time according to the current experimental equipment resource information. The front-end part of the development based on the create-react-app scaffold handles the logic of the presentation layer. And you can query the specific experimental scheduling time of each class, the specific allocation time of equipment, and the arrangement of temporary new experimental courses and other functions [22].

The main experimental equipment administrators and remote users need to apply for experimental equipment. Through this module, the experimental equipment management far can query the reservation application, use information of the experimental equipment that has been received at the current time, and give permission or rejection according to the equipment status and other information. Remote users can apply for the use of experimental equipment through this module and experimental equipment priority supply for the use of users on campus, so only allowing the equipment during the time of the reservation of nonlaboratory courses.

## 4. Results Analysis

### 4.1. Performance Results of DNN Fusion Media Literacy Algorithm

The business logic layer, as the core layer of the whole recommendation system, is mainly responsible for reading data from the data layer for movie recommendation and transmitting the recommendation results to the application layer through the data structure. The business logic layer mainly consists of two parts: real-time recommendation and offline recommendation.

**Figure 5:**
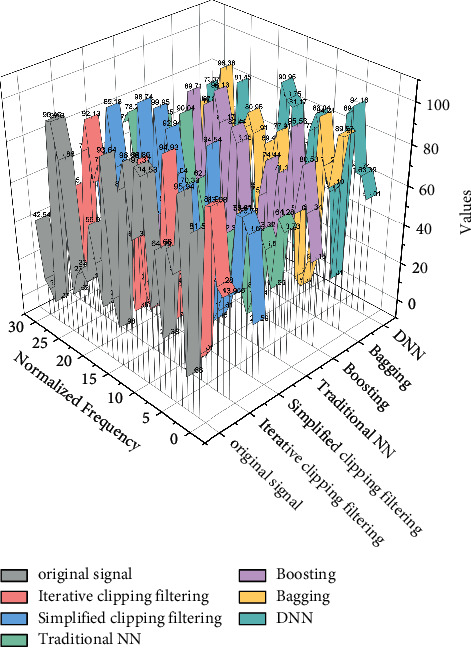
Comparison of PSD curves of each algorithm.

Since the iterative filtering algorithm and the simplified filtering algorithm both consider the impact on the spectrum when processing the signal, they do not cause interference in the out-of-band. The traditional neural network algorithm, however, reduces the filtering part of the operation, and its processing of the signal is also based on the time domain transformation, so the spectral part of the OFDM signal is not effectively controlled under the traditional neural network algorithm, which may cause the leakage of the signal spectrum and form interference to other band signals. In this paper, the proposed algorithm based on integrated learning, considering that the signal processing is performed in the time domain, which may cause out-of-band signal leakage, adds the filtering operation of the signal after the neural network processing to avoid the possibility of interference to other frequency band signals, as shown in Figure 5.

As can be seen from the figure, the PSD curve of the conventional neural network is much higher than that of the original signal processed with other algorithms, which is in line with the above inference, precisely because it does not consider the filtering results. If we want to get the corresponding test cases, the test cases are quickly designed, and the software realizes that something will go wrong. The PSD suppression effect of the two integrated learning-based algorithms proposed in this paper is like that of the iterative limit filtering algorithm and the simplified limit filtering algorithm, both of which can effectively suppress the out-of-band leakage of the signal and avoid interference with the out-of-band signal.

**Figure 6:**
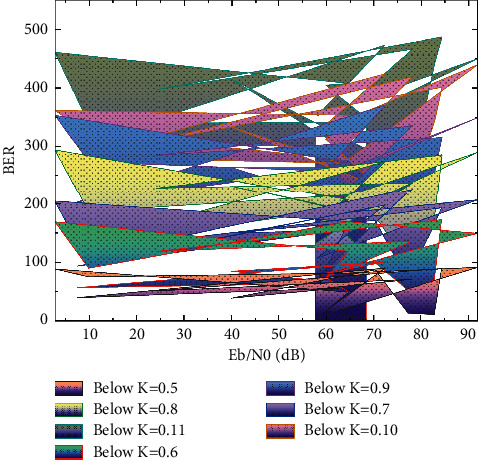
Comparison of BER curves for different *K* values.

In the process of training the neural network, the training set consists of randomly generated WLAN signals, so the cost of acquiring the training set of this algorithm is very low and easy to migrate. In this section, the minimum number of neurons for the WLAN system, i.e., 512 neurons, the learning rate of 0.001, Batch size of 100, training set size of 105 signals, Epoch times of 10, and Adam Optimizer are used as the optimization function. The variation of the loss function of the training process is shown in Figure 6. The minimum value of the loss is 0.094816 for *K* 0.05, 0.172844 for *K* 0.1, and 0.245528 for *K* 0.15, as shown in Figure 6.

The multi-task neural network algorithm is proposed based on the idea of multi-task learning, and the complexity of the network structure is further reduced by using the idea of multi-objective optimization. It has excellent suppression performance of CM, low BER, excellent suppression of out-of-band leakage, and low complexity. The final simulation results show that the proposed algorithm has advantages in OFDMCM suppression performance, out-of-band leakage suppression, and implementation complexity compared with traditional limiting algorithms and constellation expansion algorithms.

### 4.2. Analysis of Test Results of Distance Education Management System

**Figure 7:**
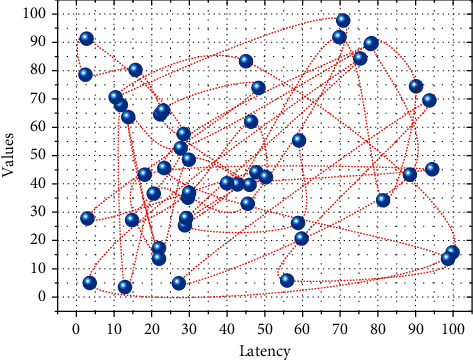
System response latency test analysis.

According to the analysis of the system test case requirements, the problems found were revised and corrected. After the revision, the problems were re-tested, and the test results showed that the problems were completely solved. Monkey testing tools are used to test and find indicators in the test process such as response time; performance testing is mainly carried out smoothly, mainly to complete the following performance testing objectives. System data operation accuracy rate is greater than 90%. The system operates stably for more than 2 hours. At the same time, to improve the generalization ability of the model, we added 100 different types of noise when designing the training set to improve the model's ability to deal with nonstationary noise. Meet 200 people using the system at the same time. System response delay is less than 3 seconds. 100 groups of experiments are used to test the interface access of 10 main programs of the system. Each group of experiments is randomly connected with the computer system response delay to obtain the system response delay test results, as shown in Figure 7.

In Figure 7, the horizontal coordinate represents the number of experiments tested, proportional to the time; the vertical axis is for the test tie response time scale in seconds, the overall smooth jitter between 0.5 seconds and 2 seconds, and the entire system response delay has a slight upward trend, the system's running time growth, fully meeting the needs of users.

In the process of long-term dependence, physiological conditioned reflex will be formed. Once someone leaves the company of sound, it will even produce physiological discomfort.

Many young people are currently obsessed with voice acting, sometimes beyond normal control. Such problems are not unique to the medium of mobile audio, as every medium has similar problems during the development of mass communication. However, the original mission of the media should not be lost in the process of development. It should play the role of media guidance, in the appropriate number of moderate standards, to provide nutrition for the development of culture and social life. You can also inquire about the specific experimental arrangement time of each class, the specific allocation time of equipment, and the arrangement of temporary new experimental courses.

**Figure 8:**
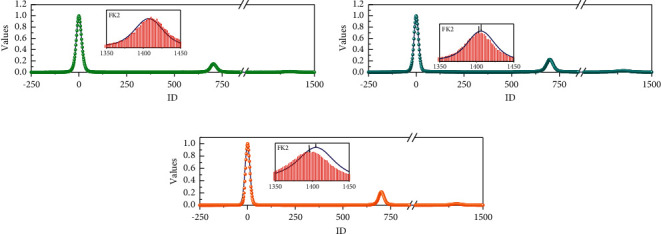
Middleware service program interface.

Users need clear permission verification in the process of using this design system, which can be based on the basic requirements of the operation, and students can select the corresponding course according to the actual situation and then manage the course grades. And in the whole grade interface, you can check your learning results, and if you have doubts about your grades, you can apply for score checking. But ordinary users cannot make changes to their grades. The system selects the corresponding course selection grades from different areas and then transmits the course selection grade management information to the database in the background, as shown in Figure 8.

The system analyzes the problem of scheduling the use of experimental equipment for campus users, combines the invocation of experimental equipment and the assignment of experimental courses, transforms the actual problem into a mathematical model, and designs the optimal scheduling model to solve the problem. The improved genetic algorithm is used to solve the optimal scheduling model, and an improved genetic algorithm based on the parent elite selection strategy is proposed to address the problem of insufficient “depth” in the generation-to-generation search of the traditional genetic algorithm. Through experimental verification, the improved genetic algorithm has obvious improvement in convergence speed and algorithm results compared with the traditional genetic algorithm and can provide a reasonable solution for the scheduling of campus users' experimental equipment.

The systematic analysis of the problem of disorderly experimental task execution faced by remote users using the equipment, the design of task assignment and system model for remote equipment scheduling, and the mathematical description and optimization objectives of the scheduling problem are presented. The task scheduling of remote devices is performed using message middleware technology, and the scheduling system architecture and dynamic scheduling algorithm are designed based on the task assignment and system model for the scheduling of experimental devices for remote users. The overall stability is between 0.5 seconds and 2 seconds, the response delay of the entire system has a slight upward trend, and the operating time of the system increases, which fully meets the needs of users. Through experimental verification, the new dynamic scheduling algorithm has a significant improvement in the total task time consumption and task success rate compared with the first-come, first-served scheduling strategy, and can meet the task scheduling requirements of remote experimental devices.

## 5. Conclusion

Through the design and implementation of the network distance learning system, reasonable suggestions and countermeasures are proposed, and the theory and technology of the network distance learning system are mainly analyzed. The design and implementation of the network distance learning system are analyzed for the basic requirements of the network distance learning system, which is the core content of the research of the article. After completing the design, to ensure the normal operation of the system, the designer should test the whole system after completing the system design. Combined with the results of the actual test, this system can meet the needs of actual distance education and can reflect the advanced and reasonable system to provide convenience for the teachers and students. In the future development of the system, to improve the efficiency of testing, automatic testing tools can be used, which can not only improve the efficiency of testing but also ensure the effectiveness of the actual testing. In conducting the current distance learning process, a more professional and more reasonable teaching system needs to be developed to achieve the smooth development of this industry. Distance learning institutions should rely on the existing teaching resources, seriously summarize the problems that exist with teaching, continuously use advanced computer technology to carry out comprehensive reform and research, eliminate the unreasonable places that existed in the past, develop a more scientific and reasonable system, avoid the problem of nonadaptation, and do a good job of popularizing the distance learning mode.

## Figures and Tables

**Table 1:** Comparison of the complexity of SCF and DNN.

Algorithm name	FFT/IFFT	Complex addition	Complex multiplication	Addition of real numbers	Real multiplication
SCF	63.39	46.61	93.66	83.35	35.87
NN	32.75	26.37	59.49	79.91	2.94
CNN	21.13	41.34	35.23	94	38.19
DNN	93.94	6.37	59.91	33.45	97.88

## Data Availability

The data used to support the findings of this study are available from the corresponding author upon request.

## References

[1] Saykili A. (2018). Distance education: definitions, generations and key concepts and future directions. *International Journal of Contemporary Educational Research*.

[2] Weller M., Jordan K., DeVries I., Rolfe V. (2018). Mapping the open education landscape: citation network analysis of historical open and distance education research. *Open Praxis*.

[3] Williamson B., Eynon R., Potter J. (2020). Pandemic politics, pedagogies and practices: digital technologies and distance education during the coronavirus emergency. *Learning, Media and Technology*.

[4] Palvia S., Aeron P., Gupta P. (2018). Online education: worldwide status, challenges, trends, and implications. *Journal of Global Information Technology Management*.

[5] Green J. K., Burrow M. S., Carvalho L. (2020). Designing for transition: supporting teachers and students cope with emergency remote education. *Postdigital Science and Education*.

[6] Yates A., Starkey L., Egerton B., Flueggen F. (2021). High school students’ experience of online learning during Covid-19: the influence of technology and pedagogy. *Technology, Pedagogy and Education*.

[7] Kara M., Erdoğdu F., Kokoç M., Cagiltay K. (2019). Challenges faced by adult learners in online distance education: a literature review. *Open Praxis*.

[8] Cacheiro-Gonzalez M. L., Medina-Rivilla A., Dominguez-Garrido M. C., Medina-Dominguez M. (2019). The learning platform in distance higher education: student’s perceptions. *The Turkish Online Journal of Distance Education*.

[9] Bozkurt A. (2019). Intellectual roots of distance education: a progressive knowledge domain analysis. *Distance Education*.

[10] Shearer R. L., Aldemir T., Hitchcock J., Resig J., Driver J., Kohler M. (2020). What students want: a vision of a future online learning experience grounded in distance education theory. *American Journal of Distance Education*.

[11] Koseoglu S., Bozkurt A. (2018). An exploratory literature review on open educational practices. *Distance Education*.

[12] Kumar V., Nanda P. (2019). Social media in higher education. *International Journal of Information and Communication Technology Education*.

[13] Kessler G. (2018). Technology and the future of language teaching. *Foreign Language Annals*.

[14] Vlachopoulos D., Makri A. (2019). Online communication and interaction in distance higher education: a framework study of good practice. *International Review of Education*.

[15] Fontana M. T. (2020). Gamification of ChemDraw during the COVID-19 pandemic: investigating how a serious, educational-game tournament (molecule madness) impacts student wellness and organic chemistry skills while distance learning. *Journal of Chemical Education*.

[16] Grant M. M. (2019). Difficulties in defining mobile learning: analysis, design characteristics, and implications. *Educational Technology Research & Development*.

[17] Bolliger D. U., Halupa C. (2018). Online student perceptions of engagement, transactional distance, and outcomes. *Distance Education*.

[18] Hamidi H., Chavoshi A. (2018). Analysis of the essential factors for the adoption of mobile learning in higher education: a case study of students of the University of Technology. *Telematics and Informatics*.

[19] Toots M. (2019). Why E-participation systems fail: the case of Estonia’s Osale.ee. *Government Information Quarterly*.

[20] Wang H. C., Chen C. W. Y. (2020). Learning English from YouTubers: English L2 learners’ self-regulated language learning on YouTube. *Innovation in Language Learning and Teaching*.

[21] Neuwirth L. S., Jović S., Mukherji B. R. (2021). Reimagining higher education during and post-COVID-19: challenges and opportunities. *Journal of Adult and Continuing Education*.

[22] Sánchez P. A., de Haro-Rodríguez R., Martínez R. M. (2019). Barriers to student learning and participation in an inclusive school as perceived by future education professionals. *Journal of New Approaches in Educational Research*.

